# Cellular mechanisms generating bursting activity in neuronal networks

**DOI:** 10.1186/1471-2202-15-S1-P182

**Published:** 2014-07-21

**Authors:** JingJing F Cannon, William H Barnett, Gennady S Cymbalyuk

**Affiliations:** 1 Neuroscience Institute, Georgia State University, Atlanta, GA, 30302, USA

## 

An open question in neuroscience is how the temporal characteristics of bursting activity are controlled by intrinsic biophysical characteristics. We present two mechanisms organized around the cornerstone bifurcation in a 3D Hodgkin-Huxley style neuronal model. This bifurcation satisfies the criteria for both the Shilnikov blue sky catastrophe and the saddle-node bifurcation on an invariant circle (SNIC) [1-3]. The burst duration (BD) and interburst interval (IBI) increase as the inverse of the square root of the difference between the corresponding parameter and its bifurcation value. The cornerstone bifurcation also determines the stereotypical transient responses of silent and spiking neurons [3]. The mechanisms presented here are based on these transient responses.

**Figure 1:**
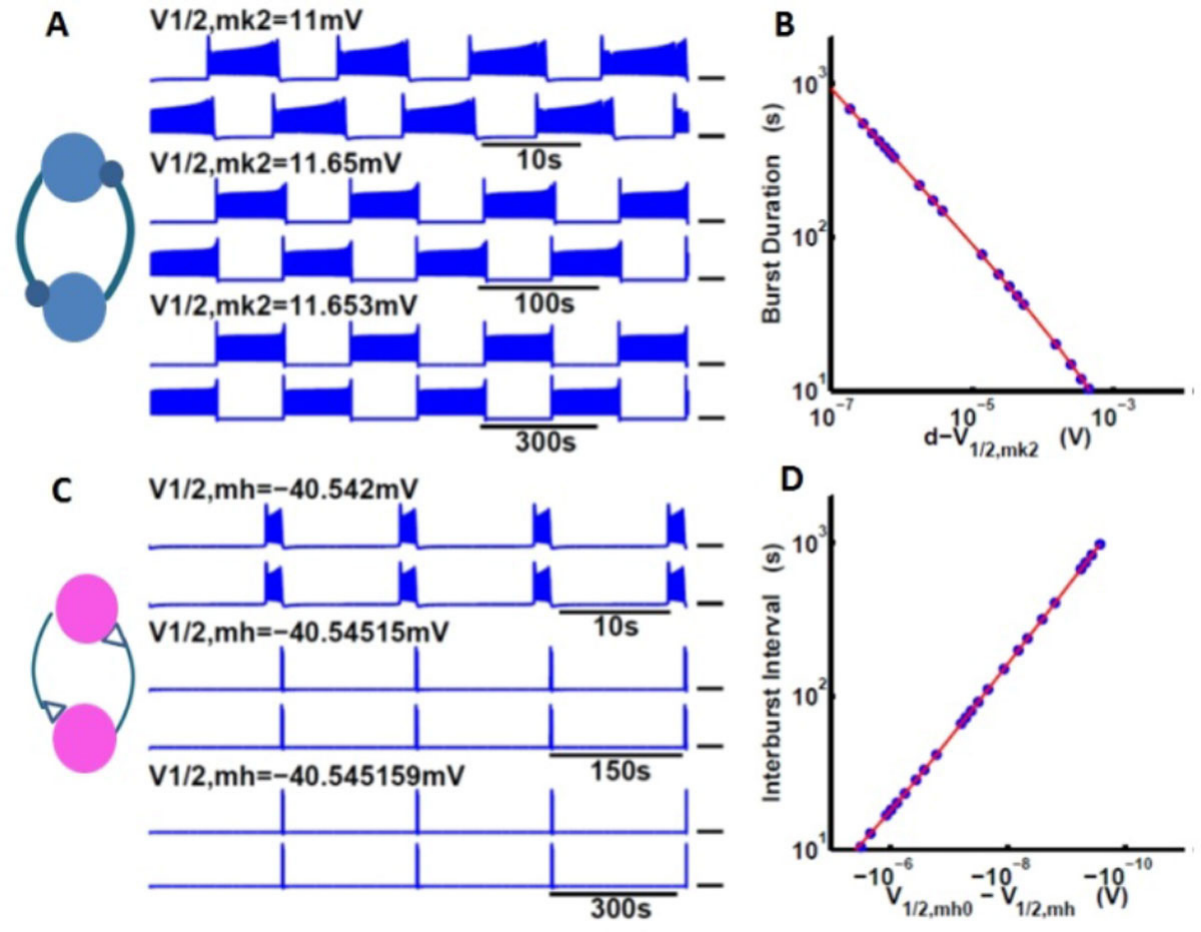
**The cornerstone bifurcation generates mechanisms controlling bursting in neural networks**. (A). Two endogenously silent, mutually inhibitory coupled neurons produce alternating bursting activity. V_1/2,mk2 _controls BDs. (B). The BDs are depicted as blue dots. The curve fit for BDs took the form b/$\sqrt{d-V_{1/2,mk2}}+\text{c}$. (C). Two endogenously spiking, mutually inhibitory coupled neurons produce synchronous bursting activity. V_1/2,mh _controls IBIs. (D).Curve fit for IBIs took the form b/$\sqrt{{\text{V}}_{1/2,\text{mh}}--{\text{V}}_{1/2,\text{mh}0}}$. The curves fitted to these data are depicted in red.

The first mechanism described half-center oscillator consisting of two intrinsically silent, mutually inhibitory neurons (Figure 1AB). These two neurons were in the silent parameter regime but close to the cornerstone bifurcation. The two coupled neurons showed anti-phase bursting activity without the release and escape mechanisms. We found that if the half-activation voltage of a non-inactivating potassium current (V_1/2,mk2_) was systematically shifted towards the bifurcation value for the saddle-node bifurcation of periodic orbits, the BDs of both neurons increased in accordance with the inverse-square-root law and exhibited linear dependence on the spike number per burst.

The second mechanism described the bursting activity of two intrinsically spiking, mutually excitatory neurons. The parameters of the neurons were in vicinity of the cornerstone bifurcation. This network exhibited synchronized bursting (Figure 1CD). Remarkably, excitatory interaction between endogenously spiking neurons essentially led to reduction of excitability of the network. When the half-activation voltage of hyperpolarization-activated current (V_1/2,mh_) was systematically shifted to the bifurcation value, IBIs of both neurons increased in accordance with the inverse-square-root law and the BDs and the number of spikes per burst stayed constant (6 spikes per burst).

This study reveals new mechanisms controlling bursting activity in small neuronal networks based on cellular properties determining transient responses of endogenously spiking and silent neurons. These mechanisms are generic and could govern the bursting regimes in rhythmic neuronal network such as central pattern generators.
